# Reinforcing Mechanisms of Graphene and Nano-TiC in Al_2_O_3_-Based Ceramic-Tool Materials

**DOI:** 10.3390/nano10091815

**Published:** 2020-09-11

**Authors:** Zhefei Sun, Jun Zhao, Xuchao Wang, Enzhao Cui, Hao Yu

**Affiliations:** 1 Key Laboratory of High Efficiency and Clean Mechanical Manufacture of MOE, School of Mechanical Engineering, Shandong University, Jinan 250061, China; sunzf@mail.sdu.edu.cn (Z.S.); wangxuchao@mail.sdu.edu.cn (X.W.); 201612688@mail.sdu.edu.cn (E.C.); 201813949@mail.sdu.edu.cn (H.Y.); 2 National Demonstration Center for Experimental Mechanical Engineering Education, Shandong University, Jinan 250061, China

**Keywords:** graphene/nano-TiC, ceramics, microstructure, mechanical properties, reinforcing mechanisms

## Abstract

Graphene and nano-TiC, which have good reinforcing effects on Al_2_O_3_-based ceramic-tool materials, are generally used as additive phases for ceramics. In this study, nine kinds of samples were sintered, to investigate the effects of graphene and nano-TiC on the reinforcing mechanisms of Al_2_O_3_-based ceramics. The experimental results indicated that adding 0.5 vol% graphene and 10 vol% nano-TiC can obtain the optimum flexural strength, fracture toughness, and Vickers hardness, which were 705 ± 44 MPa, 7.4 ± 0.4 MPa m^1/2^, and 20.5 ± 0.8 GPa, respectively. Furthermore, the reinforcing mechanisms of crack bridging, pull-out of graphene, and pull-out of nano-TiC are identified, which are contributed to improving the mechanical properties of ceramics. Meanwhile, other reinforcing mechanisms induced by graphene (graphene break, crack guiding, and 3D propagation) and nano-TiC (crack branching, crack deflection, and peeling) are discussed. These reinforcing mechanisms are coupled together, while decoupling is hard to work out. Thus, further quantitative studies of reinforcing effects of graphene and nano-TiC on Al_2_O_3_-based ceramic-tool materials are necessary to be carried out.

## 1. Introduction

Cutting-tool material, attracting much attention of researchers, is an extremely important factor in the fields of high-speed machining. Among cutting-tool materials, Al_2_O_3_-based ceramics are extensively applied due to their excellent Vickers hardness and fine thermal and chemical properties [1,2,3]. Until now, many studies have been striving to explore ways to perfect the mechanical properties of Al_2_O_3_-based ceramics. Some methods have been proposed, such as whisker toughening, phase transformation, the particle dispersion, and synergistic effects by various reinforcing mechanisms [4,5,6,7].

The addition of nanoparticles (TiC, Al_2_O_3_, SiC, TiN, ZrO_2_, etc.) is an effective method to reinforce Al_2_O_3_-based ceramics, due to their small grain size and more grain boundaries of the nanoparticles. Pezzotti et al., who explored the strengthening mechanisms of Al_2_O_3_/SiC composites, found that SiC particles tightened the grain boundary and shielded it from possible grain-boundary defects [8]. Deng et al. added TiB_2_ particles and SiC whiskers into the Al_2_O_3_ matrix and discovered that the fracture toughness was significantly increased, which was induced by the additive phases [9]. In 2004, the discovery of graphene enriched the family of reinforcing materials for ceramics [10]. At the same time, graphene attracted increasing attentions due to the large specific surface area, good fracture toughness and electrical properties, etc. Ramirez reported that the toughening mechanism of ceramic composites was the bridging effect by graphene [11]. Porwal claimed that 0.8 vol% graphene was beneficial to promote the increase of transgranular fracture during the fracture process [12]. Wang [13] and Cui [14] discussed that graphene could induce strong and weak interfaces between the ceramic matrix, which was helpful for improving the ceramics’ properties.

Many researchers have reported the synergistic effects of different toughening mechanisms, and some exploratory works have focused on the reinforcing theory [15,16,17,18]. Ceramic tools with various reinforcing mechanisms are usually conducive to improving the ceramics properties. However, at present, studies on the synergistic reinforcing effects of graphene and nano-TiC are not adequate.

The main sintering methods for ceramics are hot-pressing (HP) sintering [19,20], microwave (MW) sintering [21], and spark plasma sintering (SPS) [22]. MW furnaces are selective to sintered composites, and different composites require different microwave oven parameters. SPS has the advantages of shorter sintering time and lower sintering temperature, which are beneficial in obtaining high-density materials. However, the price of SPS equipment is expensive; thus, it is difficult to realize the industrial production. HP has the characteristics of easy operation and short holding time, etc. Moreover, it does not require the addition of a molding agent, reducing the introduction of impurities. Therefore, HP is an ideal choice for fabricating industrial ceramic composites.

At present, few studies on graphene/nano-TiC-reinforced Al_2_O_3_-based ceramic-tool materials could be found. In this study, HP was used to fabricate Al_2_O_3_-based samples, under vacuum conditions. The effects of graphene and nano-TiC on the microstructure were studied, and the optimal material composition was obtained. Finally, some reinforcing mechanisms induced by graphene and nano-TiC were analyzed.

## 2. Materials and Methods

### 2.1. Materials

**Figure 1:**
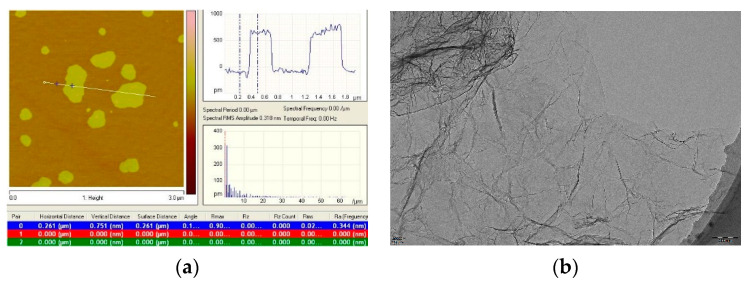
(**a**) Graphene properties from AFM, and (**b**) graphene morphology from TEM.

The materials used are briefly described as follows: Micro-Al_2_O_3_ (0.5 µm), nano-Al_2_O_3_ (0.1 µm), and nano-TiC (0.04 µm), with a purity of 99.9%, were all bought from Shanghai Chaowei nanotechnology Co. Ltd., Shanghai, China. The graphene was purchased from XFNANO Materials Technology Co. Ltd., Nanjing, China, and it had a diameter and thickness of 0.5–5 µm and 0–0.8 nm, respectively. Figure 1 shows the properties of the graphene we used. The slicer diameter and thickness of graphene were measured by using atomic force microscopy (AFM, Bruker, Santa Barbara, CA, USA). Figure 1a shows which properties are consistent with the graphene we purchased. Figure 1b presents the wrinkled and curled 2D morphology of graphene, which are helpful in reinforcing matrix materials [23]. The large specific surface area could expand the crack area of ceramics, which is beneficial to improve the fracture toughness [13].

**Figure 2:**
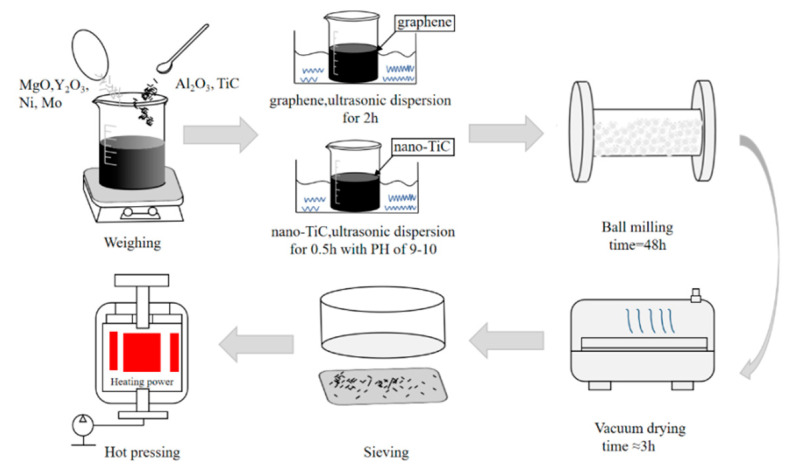
Brief experimental procedure of the composites.

MgO (Tianjin North Tianyi Chemical Reagent Factory, Tianjin, China) and Y_2_O_3_ (Shanghai Chaowei nanotechnology Co. Ltd., Shanghai, China) were used as sintering aids. Ni (Shanghai Chaowei nanotechnology Co. Ltd., Shanghai, China) and Mo (Shanghai Chaowei nanotechnology Co. Ltd., Shanghai, China) were used as metal binders. Ni has good ductility and moderate hardness, which can form a liquid phase to promote the sintering process. Adding a certain amount of Mo to Ni could significantly improve the wettability of Ni to nano-TiC. In addition, Polyvinylpyrrolidone (PVP, Tianjin Kermel Chemical Reagent Science and Technology Co. Ltd., Tianjin, China), which was an effective dispersant for graphene, was used to disperse graphene with absolute alcohol (Tianjin Fuyu Fine Chemical Co., Ltd., Tianjin, China) for 2 h. Then, nano-TiC was added into the solution of absolute alcohol and polyethylene glycol (PEG, Sinopharm Chemical Reagent Co. Ltd., Shanghai, China), and was ultrasonically dispersed for 0.5 h at the pH of 9–10. To mix the suspension evenly and grind up the large particles, we ball-milled the prepared materials for 48 h, with Al_2_O_3_ ceramic grinding balls. After the grinding, the materials were dried in a vacuum drying furnace, at 120 °C, for about 3 h. After we sieved it, the graphite mold was used to load the materials. The vacuum sintering furnace was used for HP, with a sintering temperature, pressure, and hold time of 1650 °C, 30 MPa, and 10 min, respectively. The brief experimental process of composite materials is shown in Figure 2.

As presented in Table 1, nine types of composites were fabricated under the same sintering condition by HP. The effects of different graphene contents (0–1 vol%) and nano-TiC contents (0–30 vol%) on mechanical properties of ceramic materials were studied.

### 2.2. Characterization

The sintered composites were cut into long strips that were 30 mm × 3 mm × 4 mm, by an inner circle slicer. After grinding and polishing the strips, a three-point bend tester (CTN 5150, Shenzhen Suns Technology Co., Ltd., Shenzhen, China) was used to obtain the flexural strength. Vickers hardness was calculated from a hardness tester (Model MHVD-50AP, Shanghai Jvjing Precision Instrument Manufacturing Co., Ltd., Shanghai, China), with 20 kg force, for 15 s. In addition, the fracture toughness was acquired according to the Vickers indentation method, with the following formula [24]:

(1) $$K_{IC}=0.203\times {\left(\frac{2c}{d}\right)}^{-3/2}\sqrt{\frac{d}{2}}$$

where *K_IC_*, *c*, and *d* are the fracture toughness, the average diagonal crack length, and average diagonal line length of indentation, respectively. In this paper, each group of samples was tested five times, and the average value was taken as the final result.

**Figure 3:**
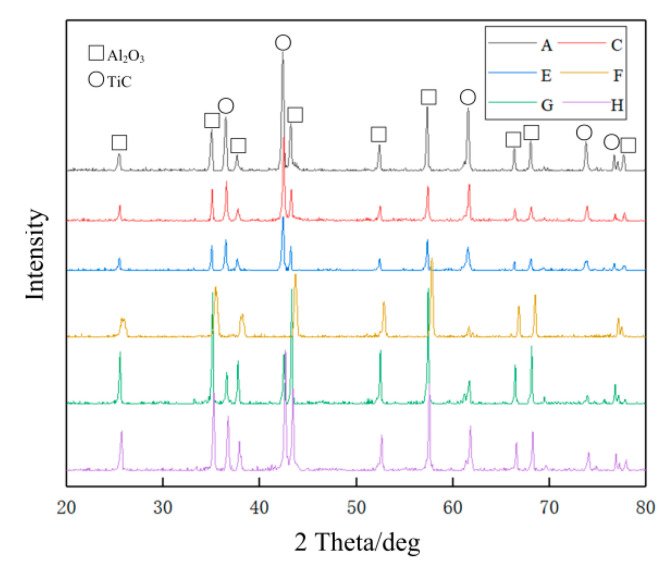
XRD spectra of composites A, C, E, F, G, and H.

As shown in Figure 3, X-ray diffraction (XRD: wavelength, 0.15418 nm; voltage, 40 kV; and current, 40 mA, Ultima IV, Rigaku, Tokyo, Japan) was used to analyze the phase composition. It can be found that the spectra of the composites are similar, which indicates that Al_2_O_3_ (corundum, Rhombo, PDF number: 00-042-1468) and nano-TiC (Cubic, PDF number: 00-001-1222) are stable and no new phases are generated at 1650 °C for 10 min by HP. Because graphene is not detected with XRD, due to its low content [14,25,26], the Raman spectrum (Model: inVia, Renishaw, United Kingdom) was used to distinguish graphene, as shown in Figure 5. After spray-gold, the morphology of the fracture surfaces and crack propagation path were observed by using SEM (SUPRA 55, Carl Zeiss Company, Oberkochen, Germany; JSM-7610F, JEOL, Tokyo, Japan). Then, energy-dispersive spectroscopy (EDS) was used to conduct element distribution.

## 3. Results

### 3.1. Effects of Graphene Content on Microstructure

**Figure 4:**
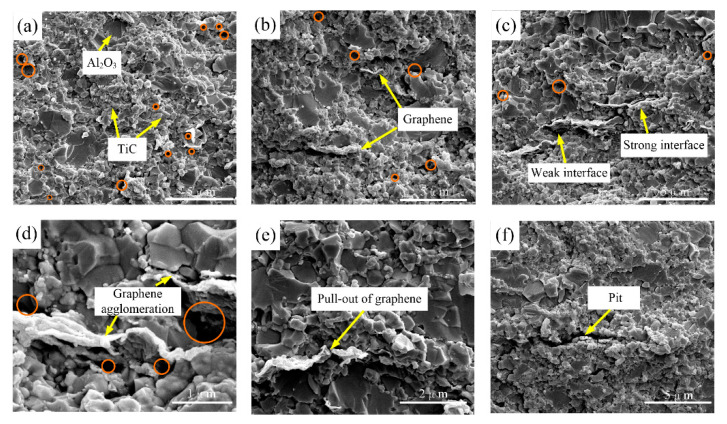
SEM morphologies of fracture surface of (**a**) composite A, (**b**) composite C, (**c**) composite E, (**d**) graphene agglomeration, (**e**) pull-out of graphene, and (**f**) pit left by graphene.

The fracture morphology of composites A, C, and E is illustrated in Figure 4. They have the same parameters, except for the graphene content. The sub-microscale grains are micro-Al_2_O_3_, and nanoscale grains are nano-Al_2_O_3_ and nano-TiC. Furthermore, graphene is embedded along grain boundaries. From Figure 4a, we see that some pores in composite A are without graphene, as marked with red circles. On one hand, pores can provide spaces for the growth of ceramic grains, which leads to abnormal grain growth and inhomogeneous grain size. On the other hand, microcracks usually occur near the pores, and they have a great adverse effect on ceramics’ properties [27]. The increase of porosity can reduce the dissipation of the fracture surface energy to a certain extent. Rice [28] claimed the relationship between porosity, *V_p_*, and fracture surface energy, *E_f_*, is given by the following equation:

(2) $$E_{f}=E_{0}\exp\left(-bV_{p}\right)$$

where *E_0_* is the fracture surface energy when *V_p_ =* 0, and *b* is the constant associated with pores. From Equation (2), it can be observed that pores are not conducive to the dissipation of the fracture surface energy. Thus, we can conclude that reducing porosity is helpful in improving the mechanical properties of ceramics. As graphene content increased, the number of pores decreased, as shown in Figure 4b,c.

It is believed that graphene is uniformly distributed along the grain boundaries, restraining excessive grain growth [29]. Uniform distribution of graphene effectively improves the densification of ceramics due to its fine properties [30,31,32]. However, with the further increase of graphene content, the agglomeration phenomenon, which is detrimental to the mechanical properties, is clearly observed. As for composite E (from Figure 4d), pores are formed between the multi-graphene and Al_2_O_3_ matrix, providing spaces for grains to grow. Different shrinkage rates and weak interface bonding strength could be the reason for this phenomenon. Furthermore, graphene agglomeration leads to a reduced “binding effect” along grain boundaries, resulting in an increase in grain size. Therefore, to obtain the fine mechanical properties of ceramic materials, the appropriate graphene content must be considered.

Pull-out of graphene (Figure 4e) and a resultant pit (Figure 4f) are observed, producing interfacial friction to consume fracture energy. Compared to nanofibers, graphene has a large specific surface area (Figure 1b), which is helpful to consume more fracture energy [33,34]. In addition, as shown in Figure 4c, an alternating distribution of strong and weak bonding interfaces induced by graphene is observed, which is beneficial in improving the mechanical properties. Reinforcing mechanisms induced by strong and weak bonding interfaces are discussed in the next section.

**Figure 5:**
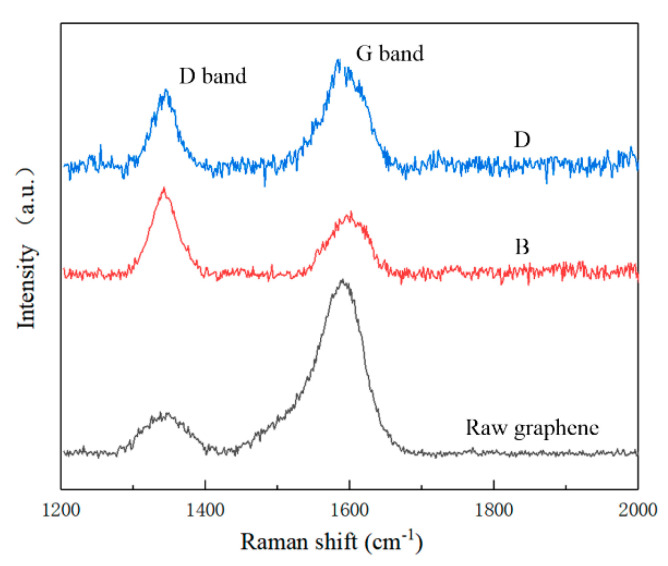
Raman spectra for raw graphene, composite B, and composite D.

As shown in Figure 5, Raman spectrum, which is an important method to characterize the crystal structure, electron band structure, and phonon energy dispersion of carbon materials with high resolution, is collected to evaluate the physical properties and defect degree of graphene. From Figure 5, we can observe two peaks for raw graphene, composite B, and composite D, at ~1348 cm^−1^ and ~1590 cm^−1^, which are D band and G band, respectively. The D band implies the disordered structure and lattice distortion, while the G band confirms the carbon–carbon band in graphene. Two characteristic peaks of graphene appear in the composites, indicating that the original characteristic structure of graphene has been retained after sintering at a high temperature and high pressure, which provides a good guarantee for the reinforcing effect of graphene on ceramic materials. The peak value of D/G of Raman intensity (*I_D_/I_G_*) are calculated, which are 0.23, 1.46, and 0.69 for raw graphene, composite B, and composite D, respectively. Interestingly, the *I_D_/I_G_* value of the composite B is higher than that of composite D, which may indicate a graphene agglomeration in composite D (shown in Figure 4d) [35,36]. Graphene agglomeration results in fewer edge numbers, thus reducing the *I_D_/I_G_* value in composite D with more graphene.

### 3.2. Effects of Nano-TiC Content on Microstructure

**Figure 6:**
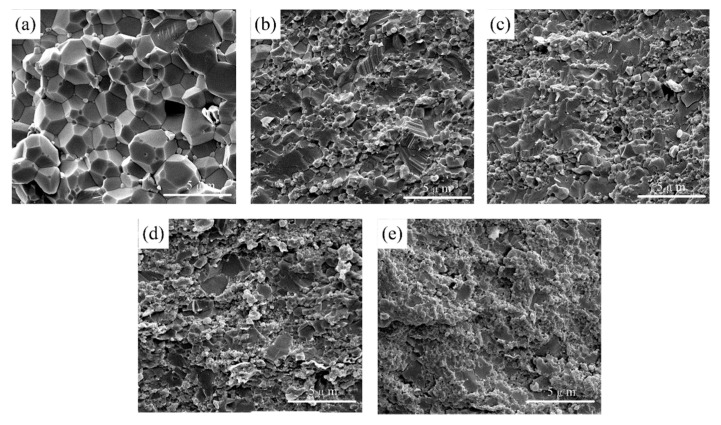
SEM morphologies of fracture surface of (**a**) composite F, (**b**) composite G, (**c**) composite H, (**d**) composite I, and (**e**) composite C.

Figure 6 shows the SEM morphology of composites F, G, H, I, and C, in which the TiC contents are 0, 5, 10, 20, and 30 vol%, respectively. Compared to microparticles or sub-microparticles, nanoparticles are much smaller, which is more conducive to preventing the matrix grains growth. Meanwhile, the obtained microstructure can be more uniform. The Hall–Petch equation [37] describes the relationship between the flexural strength and grain size, as follows:

(3) $$\sigma_{f}=\sigma_{0}+kD^{-1/2}$$

where *σ_f_* is the flexural strength, *D* is the average grain size, *k* is constant, and *σ_0_* is the strength of an infinite single crystal. The results show that the small grain size is beneficial to obtain the higher flexural strength. By comparing the fracture morphology of the sintered samples, it can be found that grain size in Figure 6a is much bigger than that of other samples. Meanwhile, some pores are seen in composite F (from Figure 6a). With the adding of low content of nano-TiC, the grain size becomes smaller. This phenomenon could be explained by the fact that nano-TiC along grain boundaries prevents the grain growth. However, nano-TiC agglomerates along grain boundaries of the ceramic matrix (Figure 6e) with the increase of nano-TiC content, making it more prone to collective spalling. Thus, the mechanical property decreases with the adding of much more nano-TiC.

**Figure 7:**
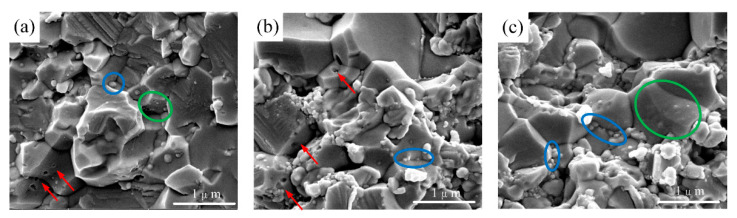
SEM morphology of (**a**) holes left by nano-TiC, (**b**) distribution of nano-TiC, and (**c**) distribution of nano-TiC. Green circles indicate nano-TiC in matrix grains, blue circles indicate nano-TiC in grain boundaries, and red arrows indicate holes left due to the peeling of nano-TiC.

As shown in Figure 7, nano-TiC is dispersed in the matrix grains (marked with green circles) and along grain boundaries (marked with blue circles). The thermal expansion coefficients of the Al_2_O_3_ matrix and nano-TiC are different at 8.6 × 10^−6^ K^−1^ and 7.6 × 10^−6^ K^−1^, respectively. Thus, small nano-TiC is enclosed by large Al_2_O_3_ grains or distributed along grain boundaries. Meanwhile, if the nano-TiC is in the crack propagation path, the crack can easily reach the nano-TiC/matrix interface. At this time, the crack will be pinned if the external force does not increase, which is the toughening mechanism of the “crack pinning”. If the external force continues to increase, the crack could pass through the grains, leading to transgranular fracture, or the crack deflection will continue to expand along grain boundaries, leading to intergranular fracture. In addition, as marked with red arrows in Figure 7a, some holes are left due to the peeling of nano-TiC, indicating a characteristic of intergranular fracture. It can be seen that some fracture surfaces are smooth and flat, indicating the existence of transgranular fracture. Thus, it can be concluded that the fracture modes are a mix of intergranular and transgranular fracture.

**Figure 8:**
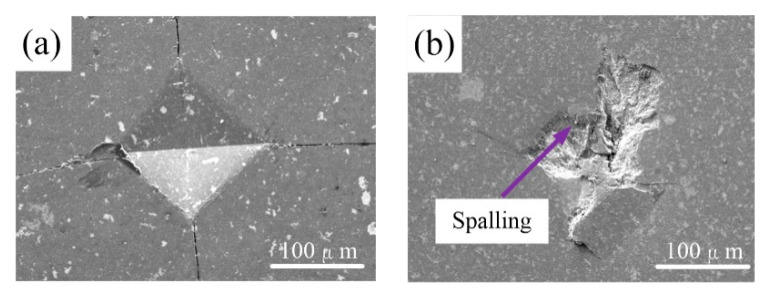
Indentation morphology of (**a**) composite H and (**b**) composite C.

Figure 8 shows the indentation morphology of composite H and composite C. It is apparent that composite H has a complete and smooth indentation morphology, whereas the surface of composite C exhibits spalling. Compared with composite C (Figure 8b), four cracks induced by indentation are clearly observed in composite H (Figure 8a). It can be concluded that the mechanical properties are decreased due to the addition of a considerable amount of nano-TiC. Similarly, Formula (2) can be used to reveal the spalling phenomenon. It is deduced that more pores are produced when more nano-TiC is added. Thus, surface spalling is apparent in Figure 8b. We can conclude that appropriate nano-TiC content is helpful in obtaining the desirable mechanical properties. However, too much is harmful.

### 3.3. Mechanical Properties

In Table 2, the value of different graphene and nano-TiC contents on mechanical properties are presented. The flexural strength increases with the increase of graphene content, from 435 ± 45 MPa for composite A to 480 ± 58 MPa for composite C, demonstrating that adding graphene can improve the flexural strength. Furthermore, the maximum of the fracture toughness is 6.5 ± 0.2 MPa m^1/2^, which shows the fracture toughness of the composites is significantly improved by adding graphene. Conversely, the Vickers hardness is not significantly improved. Nieto [38] deemed that the decreasing trend in Vickers hardness was due to the soft-phase effect of graphene. When bearing tensile force, graphene was rigid. On the other hand, the grain refinement induced by graphene slowed the decrease in hardness. The optimal sample (composite C) was obtained with 0.5 vol% graphene, which possesses comprehensive mechanical properties.

Compared with graphene, the effects of nano-TiC on the mechanical properties are quite different. The measured mechanical properties first increased and then decreased with the increase of nano-TiC content. Consequently, the optimal flexural strength, fracture toughness, and Vickers hardness are obtained at 705 ± 44 MPa, 7.4 ± 0.4 MPa m^1/2^, and 20.5 ± 0.8 GPa, respectively, with the addition of 0.5 vol% graphene and 10 vol% nano-TiC. The optimal mechanical properties are improved, as compared to those of composite A and composite E. Therefore, graphene and nano-TiC are appropriate phases to improve the properties of Al_2_O_3_-based ceramics.

### 3.4. Reinforcing Mechanisms

**Figure 9:**
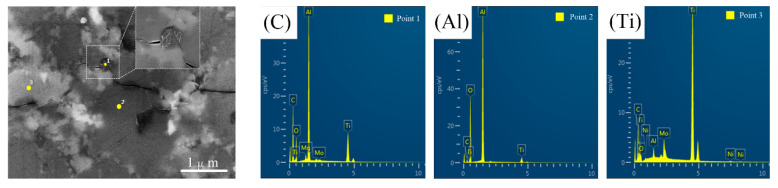
SEM morphology and element distribution of points 1, 2, and 3.

To study the reinforcing mechanisms induced by graphene and nano-TiC, indentation crack morphologies are observed by SEM. Meanwhile, EDS of three points are conducted to confirm the specific element distribution. From Figure 9, we can see that the carbon content at point 1 is evidently higher than that of other points, indicating that the substance is graphene. Similarly, the particles at point 2 and point 3 are deduced to be Al_2_O_3_ and nano-TiC, respectively.

**Figure 10:**
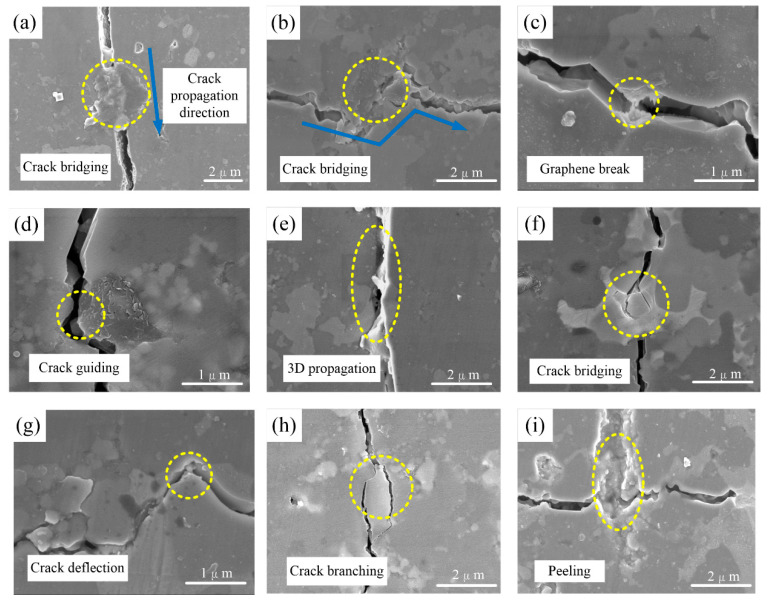
Indentation crack morphologies of reinforcing mechanisms induced by graphene and nano-TiC. (**a**,**b**) crack bridging, (**c**) graphene break, (**d**) crack guiding, (**e**) 3D propagation, (**f**) crack bridging, (**g**) crack deflection, (**h**) crack branching, (**i**) peeling.

As shown in Figure 4e and Figure 7a, graphene and nano-TiC are pulled out from the ceramic matrix under the action of loading, which is considered as a reinforcing mechanism. This reinforcing mechanism can be explained by the following formula [39]:

(4) $$\tau =\frac{\sigma_{f}d}{4L}$$

where *τ* is the interfacial shear strength, *σ_f_* is the tensile strength of the additive phase, *d* is the diameter of the additive, and *L* is the pull-out length of the additive phase. In addition, from Figure 10a–d, crack bridging, graphene break, and crack guiding are observed in the indentation surfaces after polishing. There are usually two morphologies of graphene in ceramics. One is perpendicular to the direction of the sintering pressure, while the other is parallel to the direction. Figure 10a,b presents two different patterns of crack bridging. The existence of graphene adds a “bridge”, which could limit further propagation of the crack. From Figure 10a, it can be seen that the crack propagates along a straight line, without deflection. This can be explained by the fact that the strong interface bonding strength is generated between matrix and graphene. Due to the fine ductility and strength, graphene could be a good agent to transfer stress from the matrix, which is beneficial to consume the fracture energy and strengthen the ceramic materials.

When the interface bonding strength is higher than the tensile strength, the graphene break always occurs as shown in Figure 10c. The process of graphene break could consume more fracture energy, which can be explained the fact that graphene is known as the material with higher tensile strength (~130 GPa) and Young’s modulus [40], much higher than that of the ceramic matrix. The strong bonding interface induced by graphene results in crack bridging and graphene break, while the weak bonding interface causes crack guiding (Figure 10d). It is observed that the crack propagates along the edge of graphene, which is much longer for the large 2D surface of graphene (demonstrated in Figure 1b). As studied by Walker [41], the crack deflects not only in the 2D plane but also in the 3D space, due to the introduction of graphene. It is demonstrated that graphene is more helpful in increasing the dissipation of fracture energy in the 3D deflection space than fibers or carbon nanotubes. From Figure 10e, the plane heights of the side surfaces of the graphene are significantly different, which confirms the statement to a certain degree. On the other hand, grain refinements are conducive to improving the flexural strength of ceramics, according to the Hall–Petch formula (from Equation (3)). The addition of graphene reduces the grain size, which also can strengthen ceramic materials.

As for the effects of nano-TiC, the typical reinforcing mechanism of crack bridging is observed in Figure 10f. The distance between the two sides of the cracks could not expand, due to the effect of “crack bridge”. Thus, sufficient fracture energy is consumed, and the mechanical properties are improved for ceramics. In addition, nanoparticles cause crack deflection universally, as marked with a yellow circle in Figure 10g. It is believed that the weak bonding interface and discontinuity of the microstructure [42] causes the phenomenon of crack deflection. Figure 10h illustrates a common mechanism in ceramics, namely crack branching. It can be seen that the second crack is not very long, which also helps to consume the fra**c**ture energy. However, the reinforcing effects are limited when the nano-TiC content is too high. Figure 10i presents a “river pattern” perpendicular to the crack, which is believed to be the peeling of many nanoparticles. Perhaps it is the reason for the low mechanical properties for composite E.

In conclusion, we can found the improvement of fracture toughness (via crack guiding, carck branching, crack deflection, etc.) and flexural strength (via stress transfer, grain refinement, etc.). Graphene and nano-TiC are capable of reinforcing Al_2_O_3_-based ceramic-tool materials. A strong bonding interface induced by graphene could include crack bridging and graphene break. A weak bonding interface causes crack guiding and pull-out of graphene. Of course, nano-TiC creates some of the same reinforcing mechanisms, such as crack bridging. Peeling perpendicular to the crack is observed. In addition, crack deflection and crack branching are generally found at the polishing surface.

## 4. Conclusions

Graphene/nano-TiC reinforced Al_2_O_3_-based ceramic-tool materials were fabricated at same sintering conditions in HP. The effects of graphene and nano-TiC on the microstructure, mechanical properties, and reinforcing mechanisms were discussed. The main conclusions were presented.

1. Graphene was well-dispersed and stable. However, too much graphene, which may cause some pores and weaken the “binding effect” of grains, had adverse effects on the mechanical properties. Meanwhile, PEG and ultrasonic dispersion were effective methods to disperse nano-TiC. By adding a little graphene and nano-TiC, the microstructure was much finer, which showed excellent compactness.
2. The optimal mechanical properties were obtained. After we added 0.5 vol% graphene and 10 vol% nano-TiC, the flexural strength, fracture toughness, and Vickers hardness were 705 ± 44 MPa, 7.4 ± 0.4 MPa m^1/2^, and 20.5 ± 0.8 GPa, respectively.
3. Graphene and nano-TiC synergistically induced some reinforcing mechanisms. The strong bonding interface induced by graphene caused crack bridging and graphene break, whereas the weak bonding interface caused crack guiding and pull-out of graphene. Nano-TiC also induced crack deflection and crack branching. Similarly, more pores could be found with the addition of more graphene and nano-TiC. Therefore, the appropriate amounts of graphene and nano-TiC were important for reinforcing Al_2_O_3_-based ceramic-tool materials.

## Figures and Tables

**Table 1:** Composition of composites (vol%).

Composites	Micro-Al_2_O_3_ (0.5 µm)	Nano-Al_2_O_3_ (0.5 µm)	Nano-TiC (0.04 µm)	Graphene (0.5–5 µm)	MgO (0.5 µm)	Y_2_O_3_ (0.5 µm)	Ni (0.5 μm)	Mo (0.5 μm)
A	58	10	30	0	0.5	0.5	0.5	0.5
B	57.75	10	30	0.25	0.5	0.5	0.5	0.5
C	57.5	10	30	0.5	0.5	0.5	0.5	0.5
D	57.25	10	30	0.75	0.5	0.5	0.5	0.5
E	57	10	30	1	0.5	0.5	0.5	0.5
F	87.5	10	0	0.5	0.5	0.5	0.5	0.5
G	82.5	10	5	0.5	0.5	0.5	0.5	0.5
H	77.5	10	10	0.5	0.5	0.5	0.5	0.5
I	67.5	10	20	0.5	0.5	0.5	0.5	0.5

**Table 2:** Mechanical properties of composites.

Composites	Flexural Strength (MPa)	Fracture Toughness (MPa m^1/2^)	Vickers Hardness (GPa)
A	435 ± 45	5.8 ± 0.3	15.2 ± 1.5
B	463 ± 23	6.5 ± 0.6	14.3 ± 1.7
C	480 ± 58	5.7 ± 0.2	14.8 ± 0.6
D	420 ± 26	5.4 ± 0.4	14.6 ± 1.0
E	397 ± 32	5.0 ± 0.6	13.2 ± 0.8
F	540 ± 43	6.4 ± 0.8	16.5 ± 1.9
G	607 ± 63	7.1 ± 0.3	19.9 ± 0.5
H	705 ± 44	7.4 ± 0.4	20.5 ± 0.8
I	554 ± 37	6.3 ± 0.3	15.7 ± 0.6
